# Evaluation of an Iranian Home-made *Helicobacter pylori* Stool Antigen ELISA Kit

**DOI:** 10.5812/jjm.10629

**Published:** 2014-06-01

**Authors:** Tahereh Falsafi, Paria Lavasani, Ilnaz Basardeh, Sadegh Massarrat, Zahra Landarani

**Affiliations:** 1 Department of Biology, Alzahra University, Tehran, IR Iran; 2 Digestive Disease Research Center, Shariati Hospital, Tehran University of Medical Sciences, Tehran, IR Iran

**Keywords:** *Helicobacter pylori*, Diagnosis, Iran

## Abstract

**Background:**

Current diagnosis of Helicobacter pylori infection by biopsy-based tests requires invasive sampling. Non-invasive methods such as the H. pylori stool-antigen (HpSA) test may be the best alternative for diagnosis of active infection. However, due to the presence of antigenic-diversity among the strains, various commercial tests have shown some discrepancies in different geographical-areas.

**Objectives:**

This study evaluates a homemade HpSA kit developed by using the *H. pylori* antigens from Iranian-isolates for detection of *H. pylori* in the stool of infected patients.

**Patients and Methods:**

Based on the endoscopic features and/or a rapid-urease test (RUT), 30 child and 50 adult patients, were recruited. From these candidates, three biopsies for RUT, culture and histology, and a stool-sample, were obtained. Patients were considered as *H. pylori*-positive if culture alone or RUT plus histology were found to be positive. Presence of *H. pylori* antigens in their stools was detected by the homemade HpSA test and an imported HpSA kit (Immundiagnostik, Germany).

**Results:**

Using the biopsy-based tests with RUT, histology and culture, 53% (16/30) of children were diagnosed as *H. pylori*–positive while using the imported kit 57% and the homemade kit 50% of the candidates showed positive results. Also by the biopsy-based tests, 54% of the adults were diagnosed as *H. pylori*-positive while by the homemade kit 56% showed positive results. Considering the biopsy-based tests as the gold standard, sensitivity and specificity for the imported kit was 94% and 86%, respectively, while the mean sensitivity and specificity for the homemade kit was 96% and 98%, respectively.

**Conclusions:**

The homemade kit, compared with the imported kit and biopsy-proven tests may be a valid and reliable method for determining the presence of *H. pylori* infection in Iran.

## 1. Background

*Helicobacter pylori* infect more than half of the adult and children in developing countries (1). The rate of infection in the Iranian population is, up to 80% in adults and around 50% in children (2-4). The relationship between chronic infection by *H. pylori* and stomach ulcers, duodenal ulcers and stomach adenocarcinoma has been established and to avoid these outcomes, early identification and treatment of infection is required (5, 6). The methods, which are currently used for the diagnosis of *H. pylori* infection, include culture, histology and rapid urease tests (7, 8). Although they are all accurate, yet they require invasive sampling after endoscopy. Non-invasive methods, which are based on analysis of sera or breath, also have some disadvantages.

Serological tests are unable to distinguish active from past infections since the presence of anti-*H. pylori* antibodies in serum do not necessarily indicate current infection by *H. pylori*. Serology, only has a place for epidemiological studies since after eradication, the decrease of antibody titer is very slow and remains positive in more than 30% of cases, after one year (9-11). C-13 urea breath test is a very good non-invasive test, comparable with the stool antigen test. Urease breath test has been shown to be excellent in performance, yet is not always available in routine clinical laboratories especially in developing countries (12-15). In addition, it has been associated with some disadvantages for infants and very young children, as well as patients with certain neurological disorders (12, 14, 15).

By gastric colonization, *H. pylori* are excreted into the stool and their amount in the stool may be related to the degree of stomach colonization by bacteria (16-18). Thus, their presence in the stool can be detected by *H. pylori* stool-antigen (HpSA), PCR or even culture methods (16, 18). Among stool-based tests, HpSA test has been known as a simple, relatively inexpensive and reliable assay for the diagnosis of *H. pylori* infection in adults and children (19, 20). For this purpose, various commercial tests using polyclonal or monoclonal anti-*H. pylori* antibodies have been developed (19, 21-24). However, the results of population screenings by these kits have shown some discrepancies in different geographical areas especially with tests using monoclonal antibodies (17, 19, 21, 23).

## 2. Objectives

The purpose of this study was to evaluate a homemade HpSA kit, previously developed by using the *H. pylori* antigens from Iranian isolates for the detection of *H. pylori* in the stool of infected patients. This evaluation was performed by detection of *H. pylori* antigens in the stool of child and adult control patients diagnosed as *H. pylori*-positive or *H. pylori*-negative by biopsy based tests.

## 3. Patients and Methods

### 3.1. Patients and Biopsy-Based Tests

Two groups of patients were selected for this study from which an informed consent was obtained before the endoscopy. The first group included 30 children (aged 3-14 years) recruited from patients admitted to the Children Medical Center of Tehran, who had symptoms and endoscopic features of peptic ulcer diseases and/or a positive rapid-urease test (RUT) in the endoscopy room. From these cases, two antral biopsies similar to that of RUT were obtained for culture and for histological examination and staining. Their first stool after endoscopy but before antibiotic therapy was collected and stored at -70℃ until use. The second group included 50 adults (aged 19-60 years) recruited from patients undergoing endoscopic examinations at Shariati Hospital in Tehran who had clinical symptoms of *H. pylori* infection or a positive RUT at the endoscopy room. For this group, the first stool specimen after endoscopy was also obtained and stored at -70℃ until use.

Culture of biopsy samples was performed as previously described and the grown colonies were identified by Gram-staining, oxidase, urease and PCR according to a previously described protocol (18). RUT was performed using urea broth as previously described (4). Histological examination of the biopsies was performed after Hematoxilin & Eosin and Geimsa staining. *H. pylori* density, gastritis and inflammation was graded according to standard protocols (8, 25). The status of *H. pylori* infection in the patients was evaluated according to the results of biopsy-based tests. A patient was considered as a *H. pylori*-positive control if biopsy culture alone or histology plus rapid urease test (RUT) was positive. These positives were used as the gold standards.

### 3.2. Imported HpSA Test Used in This Work

The imported HpSA test (*H. pylori* Antigen ELISA Kit, Immun Diagnostik, Germany) is based on a sandwich ELISA for detection of *H. pylori* in the stool. In this kit, a microtiter plate is coated with polyclonal antibodies specific for *H. pylori,* which bind *H. pylori* antigens from the patient sample in the first incubation step. After a washing step, the bound antigens are incubated with a biotin-labeled antibody. After a further washing step, a peroxidase-labeled streptavidin is added. Then a chromogenic substrate (TMB) is added, which produces a blue color; addition of stop solution changes this color to yellow. The intensity of the color is proportional to the amount of analyte (sample or control). 

The results are evaluated by comparison with a cut-off value, which is 0.150 at 450/620 nm and 0.190 at 450 nm. Thus, the samples with an absorbance more than 0.020 (measurement at 450/620 nm) or 0.025 (measurement at 450 nm) above the cut off-value are considered as positive and samples with an absorbance more than 0.020 (0,025) under the cut off-value are classified as negative. In this assay, absorption of the negative control at 450 nm should be less than 0.190 and that of the positive control should be greater than 0.50.

### 3.3. Initial Standardization of the Homemade HpSA Test

This test is essentially based on employment of the local *H. pylori* antigens. The local *H. pylori* strain, used for immunization of rabbits, was selected from a collection of *H. pylori* strains isolated from this region (26, 27). This selection was based on its conserved SDS-PAGE outer membrane proteins profile among strains isolated from subjects with more severe pathology of *H. pylori* infection (27). To analyze the efficiency of the immune responses produced against *H. pylori* PP23 in rabbits and to obtain its titer, the anti-*H. pylori* specific antibodies were tested with multiple *H. pylori* antigens including the local and the imported *H. pylori* antigens. For this purpose, the 96 microwell plates were coated with 5μg/ml of *H. pylori* antigens from PP23 and PP157 strains (26) and with *H. pylori* antigens from *H. pylori* Antigen ELISA Kit (Immun Diagnostik, Germany).

The ELISA assay was performed using serial dilutions of anti-PP23 antibody prepared in rabbits. In this initial study, a reaction was considered positive for antibodies to *H. pylori* if the absorbance of the reaction was > 0.5 at 450 nm and the last dilution of anti-*H. pylori* antibodies, which generated a positive reaction, was selected. This experiment was repeated and the best results were obtained from a checkerboard curve. To evaluate the minimal concentration of bacteria in stool necessary to generate a positive reaction, 1 mL portions of suspensions from strains PP23 and PP157 (26), with turbidities equivalent to those of McFarland 0.5 to 5 standards, were inoculated into the tubes containing 0.4 g of stool obtained from a negative control (a healthy subject with negative results for RUT, culture and histology tests). The stool samples, which were seeded with bacterial suspensions, were tested by the capture ELISA assay and the initial concentrations of bacteria inoculated into each stool sample was determined by CFU analysis. Next, the minimal concentration of bacteria required to generate a positive reaction was determined by comparison of OD at 450 nm between stool samples seeded with bacteria and the negative control. It was concluded that the presence of 100 bacteria in a stool sample was sufficient to generate a positive reaction. Thus, an initial optical density for the negative and the positive stools (the threshold for a negative and positive) was defined as the assay detection limits.

### 3.4. Homemade HpSA Test

This homemade HpSA test was based on a capture ELISA for detection of *H. pylori* in stools. Briefly, a 96 microwell plate (Nunc GmbH, Germany), was coated with anti-*H. pylori* antibodies prepared against membrane associated *H. pylori* antigens from the PP23 *H. pylori* strain (26, 27). These coated plates were blocked with 1% BSA in PBS (pH = 7.2) for one hour. For stool antigen preparation, a small portion of stool (0.2 g) was transferred into a vial containing 1 ml of extraction buffer by using the applicator stick, vortexed for 15 seconds and centrifuged for 10 minutes at 400 to 500 × g. One hundred microliters of the supernatant was transferred into each well and incubated at 37°C for one hour and 30 minutes. The wells were washed and incubated with the goat anti-rabbit polyvalent antibody conjugated with horseradish peroxidase (Sigma) for one hour. Next, a chromogenic substrate 3, 3’, 5, 5’ Tetramethylbenzidine, (Sigma) was added. After 20 minutes of incubation, a stop solution (1 N hydrochloric acid) was added and the intensity of the color was measured at 450 nm. The positive control was inactivated *H. pylori* antigens (in buffered solution, pH = 7.0) from PP23 and PP157 strains (26). The negative control was inactivated *Staphylococcus aureus* antigens (in buffered solution, pH = 7.0) from ATCC 25923 strain.

### 3.5. Determination of the HpSA Status and Validation of the Cut Off Value

Using the Homemade HpSA test, the stool samples from control, children and adult groups were screened for the presence of *H. pylori*. This experiment was repeated three times and the status of HpSA-positivity or HpSA-negativity was determined by comparison of the results. The stool samples from the children group were also screened by the imported HpSA kit (*H. pylori* Antigen ELISA Kit, Immun Diagnostik, Germany). The results of these screening were used for designing a threshold for positive and negative stool antigen result. So a cut-off value of 0.230 ± 0.025 at 450 nm was selected. For calculating the sensitivity and specificity of the HpSA tests, the positive HpSA results, which corresponded to the patients with a positive status (based on biopsy based tests), were considered as the true positives. The false positive or the false negative HpSA tests were those negative by biopsy-based tests but positive by HpSA or positive by biopsy-based tests but negative by HpSA tests, respectively.

## 4. Results

### 4.1. Biopsy Based Tests Results

Of the 30 children included in the first part of this study, 16 patients (53%) were classified as *H. pylori* positive by biopsy-based tests. Amongst these cases, eight had positive results for all of the three tests, four for RUT and histology, three for both RUT and culture, and one had a positive result only for the culture test. Twenty-six of these cases had endoscopic features of non-ulcer and four had the status of ulcer. Of the fifty adult patients recruited from Shariati Hospital, 27 (54%) were classified as *H. pylori* positive by biopsy-based tests. Amongst these patients, eight had positive results for all three tests, 16 for both RUT and histology, and three for both RUT and culture. The patients positive for *H. pylori* infection showed nodular and erosive gastritis, gastric ulcer, duodenal ulcer, gastroduodenal ulcer and atrophy.

### 4.2. HpSA Tests Results

The results of HpSA tests for the groups of children and adult controls are shown in Tables 1, 2, respectively. The values of sensitivity and specificity of the HpSA tests are shown in Table 3. These results demonstrate that mean sensitivity and specificity of the homemade test was 97% and 98%, respectively.

**Table 1.** Comparison Between the Results of Biopsy-Based Tests and HpSA Tests for Diagnosis of *H. pylori* Infection in the Child Control Patients

Number of Patient	Status of *H. pylori* Infection	RUT	Culture	Histology	HpSA Imported	HpSA Homemade
**m1**	negative	negative	negative	negative	negative	negative
**m2**	negative	negative	negative	negative	negative	negative
**m3**	negative	negative	negative	negative	negative	negative
**m4**	negative	negative	negative	negative	positive	negative
**m5**	positive	negative	positive	negative	positive	positive
**m6**	negative	negative	negative	negative	negative	negative
**m8**	negative	negative	negative	negative	negative	negative
**m9**	positive	positive	negative	positive	positive	positive
**m10**	negative	positive	negative	negative	negative	negative
**m11**	negative	negative	negative	negative	negative	negative
**m12**	positive	positive	positive	negative	positive	positive
**m13**	negative	negative	negative	negative	negative	negative
**m14**	positive	positive	positive	positive	positive	positive
**m15**	positive	positive	positive	negative	positive	positive
**m16**	positive	positive	negative	positive	positive	positive
**m17**	positive	positive	positive	positive	positive	positive
**m18**	positive	positive	positive	positive	positive	positive
**m19**	positive	positive	positive	positive	positive	positive
**m20**	positive	positive	positive	positive	positive	positive
**m21**	negative	negative	negative	negative	negative	negative
**m22**	positive	positive	negative	positive	positive	positive
**m23**	negative	negative	negative	negative	negative	negative
**m24**	negative	negative	negative	negative	negative	negative
**m25**	positive	positive	positive	positive	positive	positive
**m26**	negative	positive	negative	negative	negative	negative
**m28**	positive	positive	positive	negative	negative	negative
**m29**	positive	positive	negative	positive	positive	positive
**m30**	negative	positive	negative	negative	positive	negative
**m32**	positive	positive	positive	positive	positive	positive
**m33**	positive	positive	positive	positive	positive	positive

**Table 2.** Comparison Between the Results of Biopsy-Based Tests and HpSA Test for Diagnosis of *H. pylori* Infection in Control Adult Patients ^a^

Number of Patient	Status of *H. pylori* Infection	RUT	Culture	Histology of *H. pylori*	HpSA Homemade
**1**	negative	negative	negative	negative	negative
**2**	negative	positive	negative	negative	negative
**3**	positive	positive	positive	negative	positive
**4**	positive	positive	negative	positive	positive
**5**	negative	negative	negative	negative	negative
**6**	positive	positive	positive	negative	positive
**7**	positive	positive	ND	positive	positive
**8**	negative	negative	negative	ND	negative
**9**	negative	negative	negative	negative	positive
**10**	negative	negative	negative	negative	negative
**11**	positive	positive	positive	positive	positive
**12**	positive	positive	positive	positive	positive
**13**	positive	positive	ND	positive	positive
**14**	positive	positive	ND	positive	positive
**15**	negative	negative	negative	positive	negative
**16**	positive	positive	negative	positive	positive
**17**	positive	positive	positive	positive	positive
**18**	negative	negative	negative	negative	negative
**19**	negative	negative	negative	negative	negative
**20**	positive	positive	positive	positive	positive
**21**	positive	positive	positive	positive	positive
**22**	positive	positive	positive	positive	positive
**23**	positive	positive	ND	positive	positive
**24**	negative	negative	negative	negative	negative
**25**	negative	negative	negative	ND	negative
**26**	negative	negative	negative	ND	negative
**27**	negative	negative	negative	negative	negative
**28**	negative	negative	negative	negative	negative
**29**	positive	positive	positive	ND	positive
**30**	positive	positive	negative	positive	positive
**31**	positive	positive	positive	positive	positive
**32**	negative	negative	negative	negative	negative
**33**	negative	negative	negative	ND	negative
**34**	positive	positive	negative	positive	positive
**35**	positive	positive	negative	positive	positive
**36**	negative	negative	negative	negative	negative
**37**	positive	positive	negative	positive	positive
**38**	negative	negative	ND	negative	negative
**39**	positive	positive	negative	positive	positive
**40**	negative	negative	negative	positive	negative
**41**	positive	positive	positive	positive	positive
**42**	positive	positive	positive	ND	positive
**43**	negative	positive	negative	negative	negative
**44**	negative	negative	negative	negative	negative
**45**	negative	negative	negative	negative	negative
**46**	positive	positive	ND	positive	positive
**47**	positive	positive	negative	positive	positive
**48**	positive	positive	ND	positive	positive
**49**	negative	negative	negative	negative	negative
**50**	positive	positive	ND	positive	positive

^a^ Abbreviations: ND, Not determined; RUT, rapid-urease test.

**Table 3.** Sensitivity and Specificity of Immundiagnostik Imported Kit and Homemade Kit for Detection of *H. pylori* in Stool ^a^

Kit	True *H. pylori*-Positive Status	True *H. pylori* Negative Status	False Negative Status	False Positive Status	Sensitivity, %	Specificity, %
**Immundiagnostik ** ^**b**^	15	12	1	2	94	86
**Homemade ** ^**b**^	15	14	1	0	94	100
**Homemade ** ^**c**^	27	22	0	1	100	96

^a^ Sensitivity: [number of true positives/(number of true positives + number of false negatives)]; Specificity: [number of true negatives/(number of true negatives + number of false positives)].

^b^ Child patients group.

^c^ Adult patients group.

## 5. Discussion

During the last few years, non-invasive methods for the detection of *H. pylori* infection are increasingly exploited, especially those using the ELISA assays. One important advantage of these non-invasive methods is that discomfort and risk of invasive endoscopy is avoided. Among the non-invasive tests, the stool antigen test ha been approved by the authorities since it distinguishes patients with active infection from the subjects with only the precedence of *H. pylori* infection (28, 29). Several HpSA tests based on polyclonal and monoclonal antibodies against *H. pylori* antigens have been developed, which detect the presence of fecal antigens of *H. pylori,* however, their diagnostic accuracy is controversial in various geographical regions (17, 21, 24).

One reason for the presence of discrepancies among the accuracy of these assays may be the presence of significant heterogeneity among *H. pylori* strains especially among those from different geographic regions. Another problem may be the occurrence of cross reactivity with other enteric pathogens which produces uncertain results and may be due to non-adjustment of the cut off values. This possibility was excluded in the case of the HpSA assay, presented in this work. Antibody molecules are highly specific for their corresponding antigens, being able to detect one molecule of a protein antigen out of more than 108 similar molecules (30, 31). This high specificity may be responsible for the high specificity of the tests based on employment of the monoclonal antibodies. However, due to the presence of vast molecular heterogeneity among *H. pylori* strains, a monoclonal antibody prepared against a distinct antigenic determinant, would not necessarily recognize a similar one in another strain. Thus, in many cases, the sensitivities of the tests based on monoclonal antibodies are not similar, especially in geographically different regions. This inconvenience could be lower in cases of tests based on polyclonal antibodies, which have the potential to recognize numerous antigenic determinants. 

In Iran, various HpSA ELISA imported kits have been tested, however, their accuracy in the Iranian population may not be similar (3, 20, 32). Using 16 *H. pylori* positive and 14 *H. pylori* negative child patients as the gold standard group, a higher sensitivity and specificity was obtained with the Homemade HpSA kit in comparison with the Imported kit (Table 1). Although the sensitivity and specificity of this imported kit was evaluated as 97.7% and 96.3%, respectively, by the manufacturer, these values were lower in the case of Iranian *H. pylori*, suggesting its lower performance in a geographically different region, such as Iran. This is the first report evaluating and comparing the sensitivity and specificity of commercially imported HpSA ELISA kits with an Iranian homemade kit (based on local *H. pylori* antigens) for detection of *H. pylori*-specific antigens. Presence of significant molecular heterogeneity amongst Iranian and *H. pylori* strains from other regions of the world, impose the development of an HpSA ELISA kit by using local antigens. The results of this evaluation have shown that the homemade HpSA kit, compared with the imported kit and biopsy-proven tests may be a valid and reliable method for checking the presence of *H. pylori* infection in Iran.
